# Left atrial appendage thrombus; young or old? Role of CMR in definition

**DOI:** 10.1186/1532-429X-15-S1-T2

**Published:** 2013-01-30

**Authors:** Mohammed N Alnasser, Robert W Biederman, Ronald B Williams, June Yamrozik, Sahadev T Reddy

**Affiliations:** 1 Cardiac MRI, Allegheny General Hospital, Pittsburgh, PA, USA

## Background

We report an AF patient who was on Coumadin, presented with acute stroke secondary to LA/LAA thrombus and how CMR played a role in the definition of thrombus age and subsequent management of the patient.

## Methods

An 83-year-old male with history of AF, DM, cardiomyopathy presented with acute weakness and numbness of the left upper extremity. Two weeks prior to his presentation his INR reportedly ranged from 1.1 to 1.6. However, his INR level was 2.3 at the time of presentation. Upon examination, he was alert and vital signs were stable. Cardiovascular examination revealed irregularly irregular rhythm without any murmurs. Neurological evaluation revealed normal cranial nerves, mild weakness (4/5) of the left upper limb, normal motor strength over the rest of the limbs, normal sensory examination, gait and reflexes were normal.

## Results

**Figure 1:**
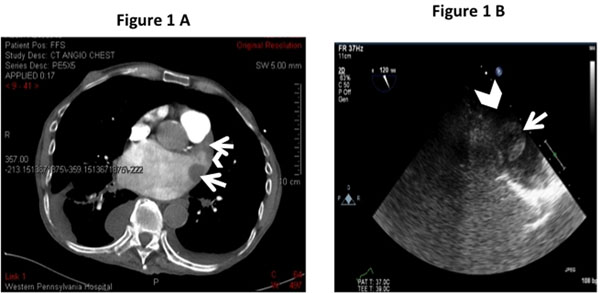
A. CT angiogram of the chest showing left atrial/left atrial appendage (arrow head) and masses (arrows) Figure 1 B. Transesophageal echocardiogram showing left atrial appendage (arrow head) and thrombus (arrow)

**Figure 2:**
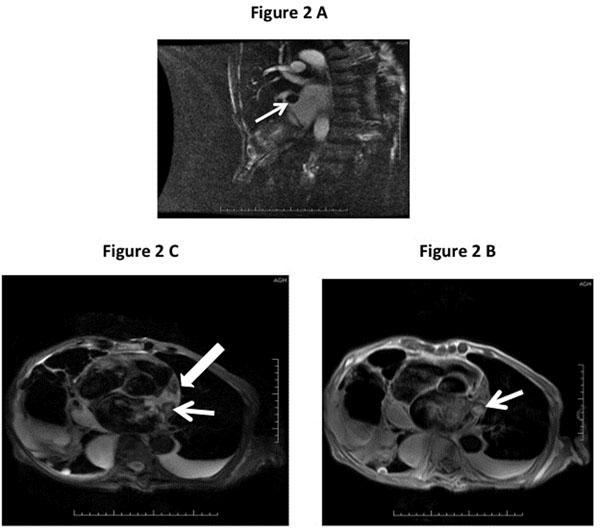
A. CMR contrast enhanced vertical long axis (VLA) showing LAA thrombus. Figure 2 B. CMR paraxial view depicting T1 weighted image of the LA and LAA with multiple thormbi. Figure 2 C. CMR paraxial view demonstrating T2 weighted image of the LA and LAA with fresh clot (narrow arrow) showing high signal intensity than the old clot with decreased central signal intensity (broad arrow).

CTA of the chest (Figure 1A) performed revealed a two-centimeter mass in the LA/LAA area and MRI of the brain showed multiple acute infarctions. TEE (Figure 1B) showed two two-centimeter irregular, potentially pedunculated masses most likely believed to be thrombus but LAA tumor or a rare myxoma was in the differential. A CMR was performed to evaluate tissue characters of the mass(s) (Figure 2A) and confirmed multiple non-mobile thrombi in the left atrial appendage with one at LA/LAA junction demonstrating high signal intensity on T1 and T2 weighted images (Figure 2B & C ) suggestive of recent clot (< three weeks old) as well as chronic clot (> four weeks old). LGE confirmed the presence of thrombus and definitively excluded tumor or myxoma.

## Conclusions

TEE provides a distinct echo window for evaluation and localization of LA/LAA thrombus compared to TTE. Detection of evolving thrombus in high-risk AF patient's LA/LAA has obvious therapeutic and clinical implications. Recent advances in CMR make it possible to differentiate tissue characters non-invasively based on water content, chemical composition and physical state of the tissue.

This approach has been popularized in our lab and was instrumental in confirming with high confidence not only the nature of the masses but also their age (in case of thrombus) rendering clinically relevant information. We were able to define unequivocally tissue characteristics (virtual histology) as well as determine the heterogeneous ages of the multiple thrombi based solely on T1 and T2 weighted CMR images. The age of thrombus as defined by CMR T2 weighted images correlated with time period where our patient's INR was sub-therapeutic and was presumed to have led to the formation of a new thrombus. We believe CMR imaging is a promising noninvasive tool in the evaluation of LA and LAA thrombus to define the age of the thrombus in addition to localization, helping to refine the intensity of treatment and to prevent further thromboembolic events which in this case was complimentary to the TEE and CT scan.

## Funding

None

